# Convex Representations Using Deep Archetypal Analysis for Predicting Glaucoma

**DOI:** 10.1109/JTEHM.2020.2982150

**Published:** 2020-05-28

**Authors:** Anshul Thakur, Michael Goldbaum, Siamak Yousefi

**Affiliations:** 1 School of Computing and Electrical EngineeringIndian Institute of Technology Mandi 231997 Mandi175005India; 2 Department of OphthalmologyUniversity of California San Diego 8784 San DiegoCA92093USA; 3 Department of OphthalmologyThe University of Tennessee Health Science Center 12326 MemphisTN38163USA; 4 Department of Genetics, Genomics, and InformaticsThe University of Tennessee Health Science Center 12326 MemphisTN38163USA

**Keywords:** Glaucoma prediction, archetypal analysis, deep archetypal analysis, artificial intelligence, machine learning

## Abstract

*Goal:* The purpose of this study was to identify clinically relevant patterns of glaucomatous vision loss through convex representation to predict glaucoma several years prior to disease onset. *Methods:* We developed a deep archetypal analysis to identify patterns of glaucomatous vision loss, and then projected visual fields over the identified patterns. Projections provided a representation that was more accurate in detecting glaucomatous vision loss, thus, more appropriate for recognizing preclinical signs of glaucoma prior to disease development. To overcome the class imbalance in prediction, we implemented a class-balanced bagging with neural networks. *Results:* Using original visual field as features of the class-balanced bagging classification provided an area under the receiver-operating characteristic curve (AUC) of 0.55 for predicting glaucoma approximately four years prior to disease development. Using convex representation of the visual fields as input features provided an AUC of 0.61 while using deep convex representation as input features improved the AUC to 0.71. Relevance vector machine (RVM) achieved an AUC of 0.64. *Conclusion:* Deep archetypal analysis representation of visual functional features with balanced bagging classification could serve as an automated tool for predicting glaucoma. *Significance:* Glaucoma is the second leading cause of worldwide blindness. Most people with glaucoma have no early symptoms or pain, delaying diagnosis in many patients until they reach late irreversible vision loss stages. In fact, about 50% of people with glaucoma are unaware they have the disease. Deep archetypal analysis models may impact clinical practice in effectively identifying at-risk glaucoma patients well prior to disease development.

## Graphical abstract

Glaucoma is the second leading cause of worldwide blindness. Most people with glaucoma have no early symptoms or pain, delaying diagnosis in many patients until they have irreversible vision loss. In fact, about 50% of people with glaucoma are unaware they have the disease. Deep archetypal analysis models may impact clinical practice in effectively identifying at-risk glaucoma patients well prior to disease development.

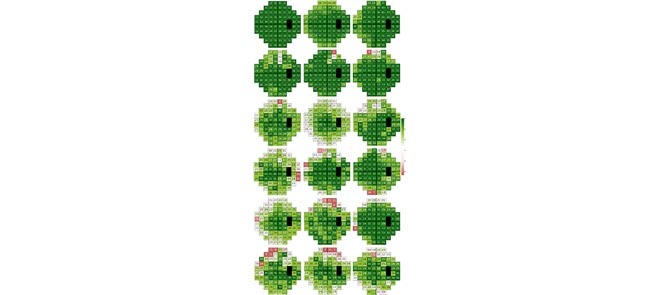

## I. Introduction

Archetypal analysis (AA) was first proposed by Cutler *et al.* [1]. However, several variations of AA has been proposed in the literature [2]–[4]. Deep AA (DAA), as a non–linear extension of the initial AA was recently proposed to address several limitations of the AA [5]–[7]. Deep AA does not rely on expert knowledge to combine relevant dimensions, learns appropriate transformations when combining features of different types and is able to incorporate additional information into the learning process.

As a proof of concept, we will deploy a particular type of DAA and show that it provides an appropriate framework for predicting ocular conditions such as glaucoma several years prior to the onset of the disease from visual field data.

Glaucoma is, in fact, a heterogeneous group of eyes diseases and the second leading causes of blindness worldwide [8]. It has multiple known risk factors including older age, African-American ethnicity, elevated intraocular pressure (IOP; fluid pressure inside the eye), and thinner central corneal thickness [9], [10]. However, subjects with these risk factors may or may not develop glaucoma as multiple other factors interact in a complex manner, making prediction of the disease in advance a non-trivial task. Moreover, glaucoma is asymptomatic and the patient is often not aware of the disease in early stages, before vision loss becomes significant [11]. Hence, any progress in predicting glaucoma early is clinically important and economically impactful.

Archetypal analysis was used to identify patterns of visual field (VF) loss of patients with glaucoma [12] and then used to detect glaucoma progression from AA-identified patterns of VF loss [13]. Recent advanced in data-driven models may also aid uncovering such hidden visual functional patterns that may lead to glaucoma and hence improve our understanding of mechanisms underlying glaucoma. These hidden patterns (of structural or functional defect) may also be helpful in developing frameworks that can predict glaucoma prior to its onset.

Currently, glaucoma-induced VF losses are mainly assessed using well-established standard automated perimetry (SAP) [14]. The Humphrey 30-2 testing system generates a map of 76 local retinal sensitivities to the light. VF map is typically used by clinicians subjectively to determine the severity of glaucoma-induced functional loss and remain as an important component of glaucoma assessment.

In this study, the authors propose a framework based on DAA of VFs for predicting glaucoma several years prior to clinical manifestation of the disease. The proposed framework provides convex representations from VFs, which is more specific and sensitive for predicting glaucoma in advance. The experimental results on a real-world glaucoma dataset signify the effectiveness of the proposed framework.

The rest of the paper is organized as follows: Section II provides literature review, section III discusses AA and deep AA, section IV explains the proposed framework, section V and is devoted to experimentation. Results and discussion are provided in section VI, and finally, conclusion is provided in sections VII.

## II. Literature Review

Most of the previous studies for identifying glaucoma have been focused on utilizing machine learning or its subclass deep learning to diagnose glaucoma (clinical signs are obvious) [15]–[18]. In fact, a significant majority of deep learning models have been centered on diagnosis because of two major reasons. First, diagnosis requires cross-sectional data, which is easier to access. Second, models typically perform better for diagnosis because disease signs are already present thus easier to identify. However, predicting the future development of the disease from baseline parameters is a challenging task because 1) access to longitudinal data prior to disease development is more challenging and 2) identifying disease preclinical signs (that are not obvious yet) is more involved.

A few studies have attempted to predict glaucoma prior to disease onset [10], [19], [20]. However, most of those studies have only utilized statistical analysis to determine risk factors that may lead to the disease. Sehi *et al.* [19] used structural features such as optic nerve head topography and retinal nerve fiber layer thickness and applied a cox hazard model to predict glaucoma. Salvetat *et al.* [10] used multivariate cox hazard models and identified several structural and functional features that can serve as risk factors for glaucoma. However, the identified risk factors through statistical analysis were imprecise for prediction of glaucoma in advance of disease onset. To address this challenge, Bowd *et al.* [20] developed a machine learning classification using a combination structural and functional features to predict glaucoma prior to onset. They utilized relevance vector machines (RVM) as a classification model. To the best of our knowledge, that is the only study that has developed a machine learning-based framework for predicting glaucoma onset from baseline parameters. However, in their study, they only used VFs, fed the VF input features to the RVM classifier while more clinically relevant representations could improve recognizing subtle (preclinical) signs of the disease. Moreover, the datasets used in all the aforementioned studies are relatively small, and generalization of results of these studies may be incomplete.

The major highlights of our study are:

### A. Clinically Relevant Unsupervised Convex Representation

**FIGURE 1.**
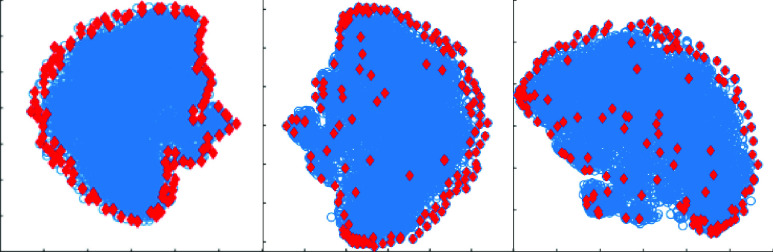
Modelling properties of 128 DAA archetypes (atoms) obtained from (left) the first layer, (middle) the third layer, and (right) the fifth layer of the DAA framework by decomposing $76-d$ VFs. Archetypes (red) and VFs (blue) were projected onto a $2-d$ space using t-SNE for visualization.

**FIGURE 2.**
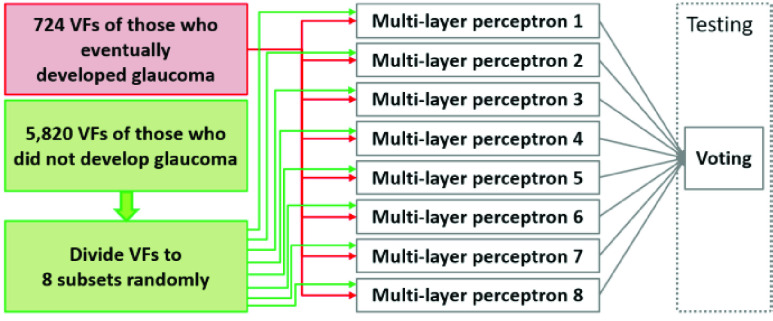
Class-balanced bagging approach used for training the proposed framework.

Since we are analyzing VF data from eyes with normal VF and normal appearing optic nerve (according to clinical standards) at the baseline (when the participants entered the study then followed for years to see when they develop the disease), using VFs directly may not provide sufficient information for predicting glaucoma. We hypothesize that there are subtle hidden patterns of VF defect that are either unknown to clinicians or challenging to detect but they may be characteristic patterns of future glaucoma development. To overcome this issue, deep archetypal analysis (DAA) and simplex projection are employed to transform VFs into unsupervised convex representations (see section III and IV) prior to be used in machine learning classifiers. DAA models generate clinically relevant patterns of VF defect (Figs. 1 and 4). These patterns have been verified by a glaucoma expert in our team (M.G.) and further used in a machine learning approach to investigate their effectiveness objectively (Figs. 2 and 5). The proposed unsupervised convex representations were obtained by decomposing VFs onto the DAA patterns. We argue that VFs of eyes that progress to glaucoma have likely particular DAA patterns of vision loss that can be used to distinguish them from eyes that did not develop glaucoma. We will show that unsupervised convex representation provides clues about the forthcoming onset of the disease, thus effective in improving glaucoma prediction.

**FIGURE 3.**
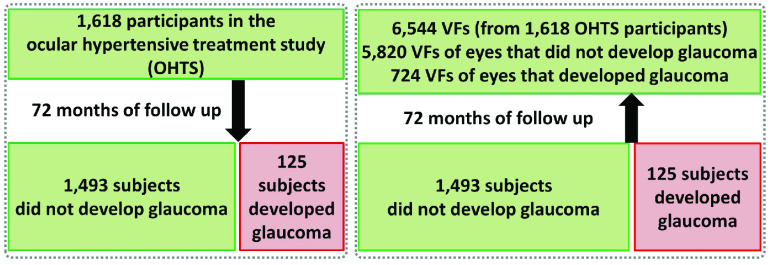
Subjects and collected visual fields (VFs) in the ocular hypertension treatment study (OHTS). Left: Participants who were selected for the study and proportion who eventually developed glaucoma. Right: VFs of participants at the baseline (start of the study). Question was that whether we can recognize hidden pattern of visual functional loss from baseline VFs that may distinguish eyes that will eventually develop glaucoma from those that will not develop the disease.

**FIGURE 4.**
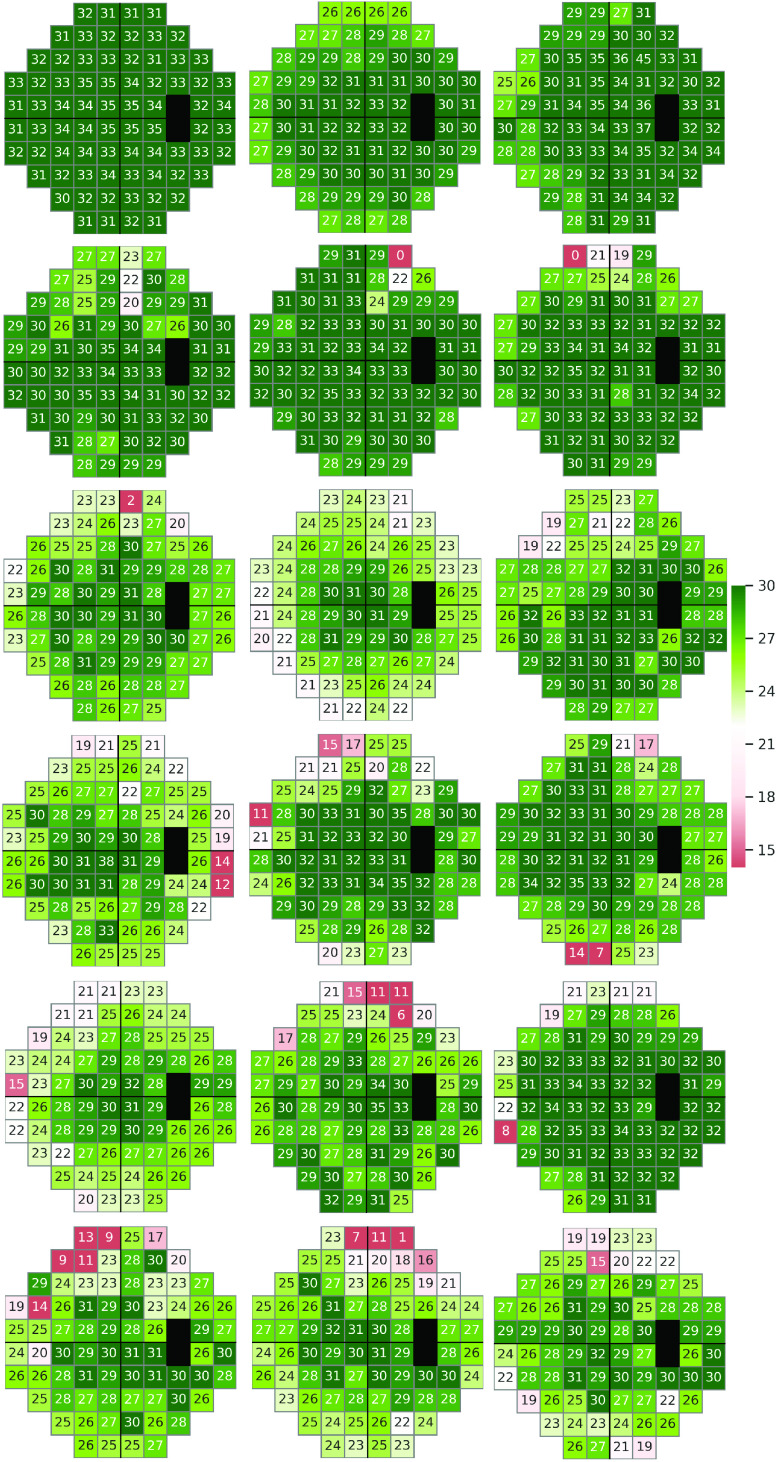
Visual field defect patterns identified by deep archetypal analysis (DAA) of VFs collected at the baseline from the Ocular Hypertension Treatment Study (OHTS) participants.

**FIGURE 5.**
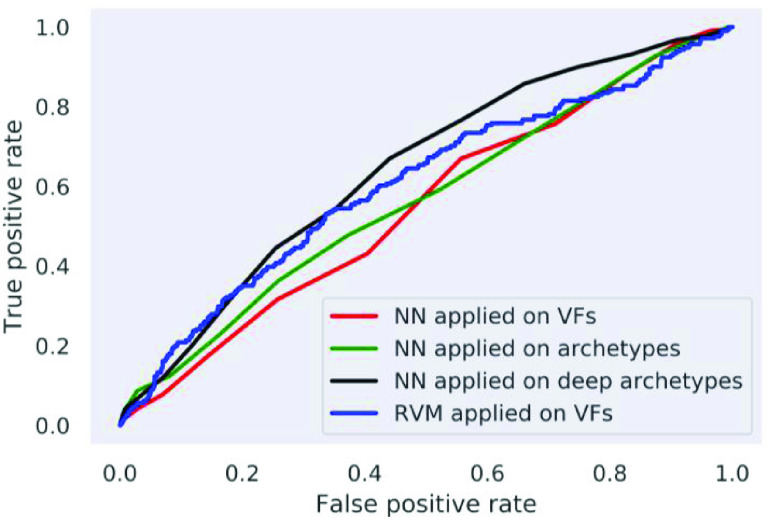
ROC curves of the using neural networks (NN) applied on original visual fields, convex representation of visual fields obtained by AA or DAA, and relevance vector machine (RVM).

### B. Class-Balanced Bagging

To overcome the inherent class imbalance in our dataset, a class-balanced bagging approach is proposed. This approach utilizes dense neural networks as base classifiers, and balanced class-examples is fed to each neural network in the training step. This approach will improve the performance by increasing the sensitivity while maintaining specificity and avoids over-fitting due to class imbalance.

## III. Archetypal and Deep Archetypal Analysis

Archetypal analysis and deep archetypal analysis both perform matrix factorization and provide appropriate means for dictionary-based learning.

### A. Archetypal Analysis

Archetypal analysis [1] is basically a matrix factorization method where a matrix, $X$ ($R^{d\times n}$), whose columns contain $d$-dimensional data points, is decomposed as $X=\mathrm { }DA$. $DR^{d\times k}$ contains $k$ archetypes that lie on extremal or convex hull of the data, and $A$ ($R^{k\times n}$) is a convex representation matrix. This implies that data points can be represented as a convex combination of archetypes. Similarly, archetypes can also be represented as a convex combination of the individual data points, that is $D=XB$, where $B$ ($R^{n\times k}$) is a convex representation matrix. Both these conditions restrict archetypes to lie only on the convex hull. Thus, archetypes present a convenient method to capture extremal properties of the input data points.

Appropriate optimization frameworks could be used to obtain archetypes $D$ from the input data points $X$ [21]:

\begin{align*}&\hspace {-2.5pc}\mathop {argmin}\limits _{B,A}{\left \|{ X-DA }\right \|_{F}^{2}} \\=&\mathop {argmin}\limits _{B,A}\left \|{ X-XBA }\right \|_{F}^{2} \\&b_{j}\in \Delta _{n},a_{i}\in \Delta _{k}\quad b_{j}\in \Delta _{n},a_{i}\in \Delta _{k} \\ \Delta _{n}\triangleq&\left [{ b_{j}\ge 0,\left \|{ b_{j} }\right \|_{1}=1 }\right],\Delta _{k}\triangleq \left [{ a_{i}\ge 0,\left \|{ a_{i} }\right \|_{1}=1 }\right] \\\tag{1}\end{align*}

Here $a_{i}$ and $b_{j}$ are columns of $A(R^{k\times n})$ and $B$ ($R^{n\times k}$), respectively. Equation 1 is non-convex as both $A$ and $B$ are unknown. Hence, the block-coordinate descent method $X$ [21] can be employed to solve this optimization problem.

Drawback: AA effectively models the convex hull of data, however, it is limited in modeling either the average or local characteristics of data. To overcome these limitations, deep archetypal analysis has been proposed by our team and others, recently [5]–[7].

### B. Deep Archetypal Analysis

Deep AA is a layered framework that performs multiple AA based factorizations on the input matrix and its subsequent factors. At the first layer of DAA, the input matrix $X$ is decomposed into an archetypal dictionary $D_{1}$ and convex-sparse representation matrix A1 using equation 1. A1 is given as input to the second layer, and is again factorized using AA to obtain dictionary $D_{2}$ and convex-sparse representations $A_{2}$. The overall factorization is: $X\approx D_{1}A_{1}\approx D_{1}D_{2}A_{2}=D_{L2}A_{2}$, where $D_{L2}$ is the DAA dictionary computed at the second layer of DAA framework. This process is repeatedly performed until reaching a user-defined depth of factorization. Hence, DAA decomposes $X$ into $L+1$ factors where $L$ is the number of layers: $X\approx D_{1}D_{2}D_{3}\ldots D_{L}A_{L}$. The factorization at each layer of DAA framework can be represented as:

\begin{align*} X\approx&D_{1}A_{1}=XB_{1}A_{1} \\[6pt] A_{1}\approx&=A_{1}B_{2}A_{2} \\[6pt] A_{2}\approx&=A_{2}B_{3}A_{3} \\[6pt] A_{L-1}\approx&=A_{L-1}B_{L}A_{L} \\[6pt] A_{L}\approx&=A_{L}B_{L+1}A_{L+1}\end{align*}

Figure 1 exhibits the data modelling capabilities of archetypes (AA atoms) and deep archetypes (DAA atoms). This figure illustrates the 2-d t-distributed stochastic neighbor embedding (t-SNE) [22] representation of 76-d VFs and AA/DAA atoms. As desired, the DAA archetypes (atoms) are modelling extremal as well as average characteristics of the data. This characteristic of DAA atoms can be attributed to the fact that dictionaries obtained at deeper layers ($L > 1$) are different convex combinations of archetypes obtained at the first layer. Since the combination of convex representations is also a convex representation [5], at any $L$’th layer ($L > 1$), a new convex representation matrix is obtained by factorizing $A_{L1}$ using AA. Hence, atoms of these deeper dictionaries can lie anywhere on the data-spread including the boundary. A DAA dictionary atom lies near the boundary if an archetype is unilaterally defining this atom in the convex combination. Similarly, if multiple archetypes have significant contribution in defining a deeper dictionary atom, it is bound to lie inside the data-spread. As a result, the DAA atoms systematically divide the data-spread into small groups, thus capturing both local and global characteristics of data.

### C. Visual Field Representation Using Archetypal and Deep Archetypal Analysis

AA/DAA is appropriate for VF data analysis because of two major reasons: 1) most of the clinically known glaucomatous patterns of VF loss lie on or near the boundary of the cloud of VF data in the initial $76-d$ space. The convex hull modelling properties of AA/DAA can identify these patterns, and hence, provides a representation that is consistent with clinical knowledge, 2) unlike many other data-dependent dictionary learning methods, AA/DAA does not project the data to any latent space. Therefore, convex representations obtained by AA/DAA are interpretable and could be clinically explained (Fig. 4).

## IV. Proposed Framework

In this section, the proposed framework for early or baseline prediction of glaucoma is described. This framework consists of two modules: feature extraction and classification as follows:

### A. Feature Extraction Using Archetypal and Deep Archetypal Analysis

This study explores how convex representation of VFs is used to provide a reliable glaucoma prediction system. The features used in the proposed framework were raw VFs, convex representation of VFs through AA, and convex representation of VFs through DAA. Deep AA is applied on training VFs to obtain the DAA dictionary ($D_{Li}$), where $L_{i}$ represents the dictionary obtained at $i$th layer of the DAA framework. Each atom of this dictionary is considered as a vertex of a high-dimensional simplex, and the remaining training and test VFs are projected on this simplex to obtain convex representations as:

\begin{equation*} y=\mathop {argmin}\limits _{y\in \Delta _{k}}\left \|{ x-D_{Li}y }\right \|_{F}^{2}\end{equation*}

such that $\Delta _{k}\triangleq \left [{ a_{i}\ge 0,\left \|{ a_{i} }\right \|_{1}=1 }\right]$. Here $x$ represents an input VF, $y$ represents its corresponding convex representation and $k$ is the number of atoms in $D_{Li}$. These convex representations are inherently sparse. We will show that the convex representations highlight the early or slight vision loss (typically due to glaucoma) in a VF that may not be captured by summary parameters of VF such as mean deviation (MD) or pattern standard deviation (PSD) that are generated by clinical instruments and widely used by clinicians.

Since no class-specific information is used to obtain DAA dictionary (unlike other AA based classification frameworks), the convex representations obtained by simplex projection is an unsupervised procedure.

### B. Class-Balanced Bagging for Classification

Like most of the datasets in real-world healthcare settings, our dataset was imbalanced as the number of subjects (who developed glaucoma at the end of the study) were significantly lower than the number of subjects who did not develop the disease. Most of the strong classifiers such as support vector machines and neural networks could be highly affected by class imbalance leading to biased towards the class with higher number of samples with a high missed detection rates. To overcome this issue, we developed a bagging-based approach where each individual classifier, a feed-forward neural network, was fed with class-balanced training examples, as illustrated in Figure 2.

In the training step, the framework divides the samples in the class with larger number of samples (those who did not develop glaucoma; called negative group) into smaller non-overlapping subsets such that the number of samples in each subset were almost equal to the number of positive class (those who eventually developed glaucoma). Each subset of negative and all the positive examples were fed to a neural network (multi-layer perceptron with similar parameters) to learn the discrimination between two classes. Therefore, each neural network was trained on the (same) positive examples but different subsets of negative examples. It is worth noting that this framework is different from traditional bagging approach where each example has similar likelihood of being selected for training in any of the eight classifiers. During testing, each neural network was considered as an independent classifier, and a majority voting rule was applied on individual predictions to obtain the final prediction.

## V. Experimentation

### A. Datasets

The ocular hypertension treatment study (OHTS) was a prospective, multi-center (across 22 centers in the US) investigation that sought to prevent or delay the onset of VF loss in patients at moderate risk of developing glaucoma [9].Unlike most of other datasets that collected glaucoma risk factors retrospectively, in the OHTS, risk factors were measured at the baseline prior to the onset of disease and afterwards routinely. Hence, the OHTS dataset allows the development and testing of a robust glaucoma prediction framework such as the framework that we developed.

A total of 1,618 subjects with elevated IOP but normal appearing optic disc (structure) and normal VF (function) were followed for about six years. The demographic parameters, clinical information and VFs were collected every six month. After about 72 months, 125 participants developed glaucoma (187 eyes) while 1,493 did not develop the disease based on VF assessments (Fig. 3). For each participant, at least two or three reliable VF tests (< 33% false positives and false negative, and < 33% fixation loss errors, according to initial OHTS study criteria) were collected by Humphrey (Carl Zeiss Meditec, Dublin, California) full threshold using the SITA Standard 30-2 procedure (covering central 30 degree of VF) at the baseline (start date of each participant in the clinical trial). Two OHTS certified readers carefully examined follow up VFs and when they identified VF abnormality, they recalled subject for re-testing to confirm the abnormality. Glaucoma onset was further confirmed by an independent endpoint committee (see [9] for more details).

In this paper, 7,248 VF tests collected only at the baseline were included for the downstream analysis (Fig. 3). We used full threshold values at each VF test location. We analyzed only VFs that were identified as normal by OHTS certified VF readers. We then hypothesized that there maybe subtle VF defect patterns in the VFs of those eyes that developed glaucoma several years later that either were missed by clinicians or were unknown to clinicians. Our aim was thus to identify those subtle VF defect patterns and show their effectiveness in predicting glaucoma well ahead of time.

### B. Training and Testing Classifiers and Comparison

10-fold stratified cross-validation was utilized for computing the accuracy in terms of the area under the receiver operating characteristics (AUC) for neural networks applied on raw VFs, convex representation through AA, convex representation through DAA, and relevance vector machine (RVM) applied on raw VFs. As discussed in Section II, the only machine learning based method for glaucoma prediction, known to the authors, was RVM [20], which was compared against the proposed frameworks. Since the dataset used in this method is publicly unavailable and part of the features utilized in this method are unavailable for all the subjects in OHTS dataset, the authors have only used their proposed classification method (RVM classifier) to provide a fair comparison.

### C. Parameter Setting and Performance Metric

All the parameters such as number of dictionary atoms (archetypes), the number of layers in DAA, the number of nodes and layers in neural network were selected such that the model provides an optimal average performance on the cross-validation data. More specifically, these parameters were selected based on an extensive grid search to provide maximum area under the ROC curve (AUC) and least missed detection rate. The proposed framework utilized 128 dictionary atoms and seven factorization levels in DAA framework. Each neural network consisted of a single hidden layer with 200 neurons. The Adam optimizer with a fixed learning rate of 0.0001 was used to train each neural network. For class-balanced bagging, the negative class was divided into eight subsets, and hence, the proposed framework was an ensemble of eight different neural networks. Gaussian kernel with a width of 0.9 was used in the baseline method for training the RVM. Similar to the proposed framework, parameter of RVM also were selected using a grid-search on the cross-validation data. The parameters of neural networks in all the experiments were kept the same. The DAA, MLP and AUC performance metrics were implemented in Python using scikit-learn library. RVM was implemented in Matlab because there was no implementation in Python. Statistical analyses were performed in R.

### D. Visual Field Defect Pattern Recognition

We applied DAA on all baseline VFs to identify hidden patterns of (glaucomatous) VF loss. We first identified 128 deep archetypes that were prevalent in the data. We then excluded the archetypes that had significant correlation with other archetypes. Figure 4 shows 18 DAA VF defect patterns that were identified from baseline VF data of the OHTS participants. These patterns were assessed subjectively by a glaucoma expert to identify its clinical relevance (M.G.). Top-left pattern was identified as a normal pattern while other patterns were identified as patterns of (early) VF loss. To further assess the effectiveness of the models, the DAA archetypes were fed to an NN classifier and was compared against raw VFs and classical AA archetypes (Fig. 5). Although glaucoma experts had not identified any suspicious glaucomatous patterns of VF loss in subjective evaluation of the baseline VF data of the OHTS participants, we suggest these subtle VF defect patterns as possible risk factors (signs) of glaucoma development several years in advance of clinical manifestation of disease signs (Fig. 5).

## VI. Results and Discussion

Out of 7,248 VF tests collected at the baseline, 6,544 VFs were labeled as both “reliable” and “normal”, in which 724 VFs corresponded to eyes that eventually developed glaucoma (over approximately six years of follow up) and 5,820 corresponded to eyes that did not develop glaucoma over the course of OHTS study (Fig. 3). The mean age (standard deviation; SD) of subjects in the normal and (converted to) glaucoma groups were 55.7 (9.6) and 58.8 (9.0) years, respectively (p < 0.001). Approximately 42% of subjects in the normal group were male while 56% of the subjects in the (converted to) glaucoma group were male (p < 0.001). Mean IOP of eyes in the normal and (converted to) glaucoma groups were 24.8 (2.9) and 26.1 (3.3), respectively (p < 0.001).

Visual field testing through standard automated perimetry (SAP) remains a gold standard for glaucoma assessment. Therefore, recognition of (glaucomatous) VF defect patterns is critical for diagnosis, severity identification, and therapy adjustments based on the type of defect [23]. However, manual classification of glaucoma through VFs requires significant clinical training and more importantly is labor intensive and highly subjective with limited agreement even among glaucoma specialists [24], [25]. Thus, any approach that can automatically identify early patterns of VF loss can impact glaucoma management.

Several researchers, including us, have used unsupervised learning to discover (glaucomatous) patterns of VF loss [12], [13], [26]–[29]. We have extensively used Gaussian mixture modeling (GMM) to discover patterns of VF loss and to identify glaucoma progression along those GMM-identified patterns [26]–[29]. Other teams have used classical AA for such goals [12], [13]. However, here we introduced a deep archetypal approach that identifies patterns of VF loss that are clinically more relevant (subjective evaluation) and patterns that may serve as signs of early glaucoma development (Fig. 4). We used 128 DAA patterns as input features to the classifier for predicting glaucoma. In fact, we investigated other numbers of patterns and obtained the optimum accuracy with 128 patterns. While these DAA patterns maximized the accuracy of glaucoma prediction, overlap among these patterns was significant when assessed visually. Therefore, a follow up study is warrant to provide a set of mutually exclusive DAA patterns for glaucoma assessment. To provide a fair comparison, we used 128 classical AA patterns as was used in DAA assessment. We used the implementation of Chen *et al.* for AA [21] and used the implementations in [5], [7] for DAA analysis.

While these patterns were not obvious to glaucoma experts before, DAA analysis identified these patterns as (possible) signs of future glaucoma development, which was confirmed further by objective analysis (Fig. 5, black curve with AUC of 0.71).

To avoid any bias due to multiple VF tests from same eyes of subjects, we accounted for correlation between tests and eyes of same subjects using a nested structure in generalized estimating equation (GEE) [30]. To account for multiple VFs from same eyes in training and testing of machine learning models, we selected the training and testing folds based on subjects rather than eyes or VFs.

Figure 5 illustrates the ROC curves of the proposed framework compared against RVM [20], classical AA approach, and raw VFs. The AUC of applying NN on DAA representation of VFs was 0.71 while the AUC of applying NN on classical AA representation of VFs and raw VFs were 0.61 and 0.55, respectively. The AUC of RVM [20] was 0.64. In fact, DAA provided a representation of VFs with significantly higher AUC than classical AA and original VFs in predicting the future development of glaucoma on both cross-validation and held-out datasets (statistical p < 0.001). This highlights that the deep convex representation, obtained by simplical projection, is more discriminative than the input original VFs as well AA and classical RVM.

At first, it may seem that an AUC of 0.71 is low compared to several approaches for identifying glaucoma with higher accuracy. While from statistical perspective this may seem a valid argument, from clinical perspective, the story is different. Predicting glaucoma from baseline VFs approximately five years prior to the disease development is a very challenging task. In fact, relatively, the easiest task would be performing automatic diagnosis, which means clinicians already have observed clinical signs of the disease however, in prediction there is no clinical sign and one would need to identify pre-clinical hidden patterns of the disease.

This study was conducted on VF tests with Humphrey 30-2 pattern. Other studies using VFs with Humphrey 24-2 or central 10-2 patterns may shed light on the effectiveness of DAA in predicting glaucoma. Nevertheless, VF testing is subjective, time-consuming and presenting a significant degree of variability. Therefore, future studies could investigate the role of structural data such as fundus photographs or optical coherence tomography (OCT) data in predicting glaucoma prior to disease onset.

## VII. Conclusion

In this paper, deep archetypal framework was developed to effectively predict glaucoma several years prior to disease manifestation. The framework utilizes simplex projections to obtain unsupervised convex representations of VFs. We showed that these convex representations are clinically meaningful and more discriminative than raw VFs or other classical VF analysis approaches. To overcome the class-imbalance, the proposed framework utilizes a class-balanced bagging approach. As a proof of concept, OHTS glaucoma clinical trial dataset was used to assess the effectiveness of approach for early glaucoma prediction.

Experimental results signified that a system of deep archetypal representation and class-balanced bagging improved predicting glaucoma development from baseline measurements several years prior to disease development. Future work with independent datasets may be required to verify the findings of this study.
